# Structural asymmetry and discrete nucleic acid subdomains in the *Trypanosoma brucei* kinetoplast

**DOI:** 10.1111/j.1365-2958.2007.05749.x

**Published:** 2007-06-01

**Authors:** Eva Gluenz, Michael K Shaw, Keith Gull

**Affiliations:** Sir William Dunn School of Pathology, University of Oxford South Parks Road, Oxford OX1 3RE, UK.

## Abstract

The mitochondrial genome of *Trypanosoma brucei* is contained in a specialized structure termed the kinetoplast. Kinetoplast DNA (kDNA) is organized into a concatenated network of mini and maxicircles, positioned at the base of the flagellum, to which it is physically attached. Here we have used electron microscope cytochemistry to determine structural and functional domains involved in replication and segregation of the kinetoplast. We identified two distinct subdomains within the kinetoflagellar zone (KFZ) and show that the unilateral filaments are composed of distinct inner and outer filaments. Ethanolic phosphotungstic acid (E-PTA) and EDTA regressive staining indicate that basic proteins and DNA are major constituents of the inner unilateral filaments adjoining the kDNA disc. This evidence for an intimate connection of the unilateral filaments in the KFZ with DNA provides support for models of minicircle replication involving vectorial export of free minicircles into the KFZ. Unexpectedly however, detection of DNA in the KFZ throughout the cell cycle suggests that other processes involving kDNA occur in this domain. We also describe a hitherto unrecognized, intramitochondrial, filamentous structure rich in basic proteins that links the kDNA discs during their segregation and is maintained between them for an extended period of the cell cycle.

## Introduction

Kinetoplastid protozoa possess a single mitochondrion that forms an extended tubular structure and contains within it an unusually organized mitochondrial genome, segmented into thousands of circular molecules of two size classes, termed mini- and maxicircles (Shapiro and Englund, 1995; Lukes *et al*., 2002). In the medically important African trypanosome, *Trypanosoma brucei*, the mini- and maxicircles form a topologically interlocked network, tightly packed into a disc-like structure called the kinetoplast, which is always positioned at the base of the flagellum. Maxicircles (22 kb) are present in a few dozen identical copies and contain DNA sequences that encode mitochondrial proteins, rRNA and some guide RNAs. The guide RNAs direct processing of maxicircle transcripts, which occurs by a complex process of RNA editing involving addition or deletion of uridine nucleotides (Benne *et al*., 1986; Stuart *et al*., 2005). Minicircles (1 kb), which encode the majority of guide RNAs are present in thousands of copies and heterogeneous in sequence. Accurate replication of this complex concatenated network of kinetoplast DNA (kDNA), and continuous faithful inheritance of a full complement of mini- and maxicircles to daughter cells requires specialized mechanisms that are still unclear (Liu *et al*., 2005).

Work on *Crithidia fasciculata* and *Leishmania tarentolae* (Shapiro and Englund, 1995; Morris *et al*., 2001; Liu *et al*., 2005) has established a model of kDNA network replication where minicircles are released from the network by topoisomerase-mediated decatenation and translocated into the space between the kDNA disc and the mitochondrial membrane nearest the flagellum, termed the kinetoflagellar zone (KFZ) (Drew and Englund, 2001). The replication of free minicircles occurs unidirectionally via θ-structure intermediates (Englund, 1979). After replication, gapped daughter minicircles are attached to the periphery of the network at antipodal sites (Simpson and Simpson, 1976; Ferguson *et al*., 1992; Robinson and Gull, 1994). These antipodal sites contain a number of enzymes that catalyse the final steps of minicircle replication, including structure-specific endonuclease (SSE1), DNA ligase kβ, DNA polymerase β and topoisomerase II (Melendy *et al*., 1988; Ferguson *et al*., 1992; Engel and Ray, 1999; Sinha *et al*., 2004; Downey *et al*., 2005). Maxicircles replicate via θ intermediates (Carpenter and Englund, 1995), but apparently remain linked to the network during replication. It is still an open question how separation of the DNA network is achieved mechanistically and whether there is a physical structure on which this is orchestrated.

However, a structural and functional link between the kinetoplast itself and the basal body provides the mechanism by which replicated kinetoplasts are segregated to different daughter cells (Robinson and Gull, 1991). The kinetoplast is replicated only once per cell cycle, in precise temporal co-ordination with other events of cell division (Woodward and Gull, 1990). Detailed ultrastructural studies have identified a tripartite attachment complex (TAC) that physically connects the kinetoplast to the basal body (Ogbadoyi *et al*., 2003). The TAC consists of (i) exclusion zone filaments that run from the proximal end of the basal body to the outer mitochondrial membrane, (ii) a specialized region of mitochondrial membrane resistant to detergent extraction and (iii) unilateral filaments between the inner mitochondrial membrane and one face of the kDNA disc. In addition to maintaining a physical link between kinetoplast and flagellum, the unilateral filament structure may also facilitate the ordered replication of the kDNA network (Ogbadoyi *et al*., 2003).

Given the events surrounding kDNA replication, segregation, transcription and RNA editing there is a need to develop descriptions of kinetoplast nucleic acid and protein component locations at the electron microscopic level of resolution. Cytochemical techniques have been instrumental in defining functional domains within the cell nucleus (Moyne, 1980; Fakan, 2004). Bernhard's EDTA regressive staining method differentiates between ribonucleoprotein and deoxyribonoucleoprotein particles (Bernhard, 1969). The *Trypanosoma cruzi* kinetoplast was among the original test materials used by Bernhard to demonstrate the specificity of this method to a wide range of DNA containing structures, but observations on kinetoplast substructure were not reported (Bernhard, 1969). Ethanolic phosphotungstic acid (E-PTA) staining of glutaraldehyde fixed cells specifically contrasts basic proteins, and has been used for example to analyse the ultrastructure of synapses, chromosomes and the *T. cruzi* flagellar attachment zone (Bloom and Aghajanian, 1966; Sheridan and Barrnett, 1969; Rocha *et al*., 2006).

Here we use these techniques to examine structural domains in the *T. brucei* kinetoplast and relate them to the current model of kinetoplast replication and function. We found that DNA and basic proteins are distributed asymmetrically around the kDNA disc, and that their localization is restricted to two specific domains: the antipodal sites and a subdomain of the KFZ. The KFZ is the site where the unilateral filaments are located. Differential cytochemical reactivity within subdomains of the KFZ enabled us to distinguish two types of unilateral filaments: the inner unilateral filaments are confined to a ∼50 nm wide subdomain of the KFZ adjacent to the disc. A distinct set of outer unilateral filaments extends from the inner filaments to the membrane. The presence of DNA in a KFZ subdomain, inferred from the EDTA bleaching of the inner unilateral filaments, is consistent with, and provides independent ultrastructural evidence for the model that minicircles are released from the network in a vectorial manner (Drew and Englund, 2001). Unexpectedly, however, our data suggest that DNA and basic proteins reside in the KFZ throughout the cell cycle, and that that this domain may be the site of additional processes involving kDNA. Our studies also revealed novel structures associated with the segregation of kDNA discs. However, we found no evidence of discrete RNA concentrations within the kDNA network.

## Results

**Fig. 1:**
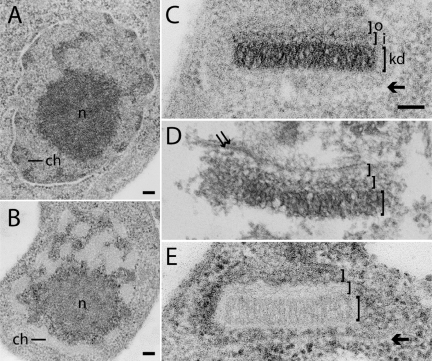
Detection of DNA containing domains by EDTA bleaching A. Section through a *T. brucei* nucleus stained with uranyl acetate. B. Treatment with EDTA for 15 min removes uranyl acetate from DNA, resulting in a bleached appearance of chromatin (ch). RNA containing structures including the nucleolus (n) are not bleached. C. Section through a kinetoplast stained with uranyl acetate. D. A kinetoplast that was extracted with detergent. E. Kinetoplast treated 15 min with EDTA. A large bracket marks the kDNA disc (kd). Small brackets indicate the extension of the inner unilateral filaments (i) and the outer unilateral filaments (o). The kDNA disc and the inner unilateral filaments are strongly contrasted with uranyl acetate, and completely bleached by EDTA treatment, indicating an asymmetric distribution of DNA around the disc. Because the material shown in C and E was not postfixed in osmium tetroxide, membranes are not specifically contrasted, but their position can be inferred from a gap between cytoplasmic ribosomes and the mitochondrial matrix (arrow). A double arrow marks the differentiated mitochondrial membrane in D. All images are oriented so that the basal body side of the kDNA disc is facing up. Scale bars represent 100 nm.

We used Bernhard's EDTA regressive stain to analyse the distribution of DNA in the kinetoplast of *T. brucei* by transmission electron microscopy. Figure 1 illustrates the specific differentiation of DNA-containing structures achieved by this method, which rapidly removes uranyl acetate stain from deoxyribonucleoprotein but not ribonucleoprotein particles (Bernhard, 1969; Moyne, 1980). In the nucleus, normal electron microscopic staining of sections with uranyl acetate produced the expected dark staining of chromatin and the nucleolus (Fig. 1A). Cytoplasmic ribosomes are also stained, albeit less intensely than nuclear chromatin and kDNA. In EDTA-treated sections however (Fig. 1B), large areas of the nucleus have completely lost their electron density and appear ‘bleached’, while the nucleolus and cytoplasmic ribosomes retain contrast. At higher magnification, details of kinetoplast ultrastructure are clearly visible. Figure 1C–E show sections through the kinetoplast cut parallel to the longitudinal axis of the kDNA disc, oriented such that the basal body side is facing up. Stained with uranyl acetate (Fig. 1C), the kDNA disc appears as a characteristic striate structure of densely stained fibres that run parallel to the longitudinal axis of the kDNA disc. The measured width of the *T. brucei* disc was 96 nm (±7.5, *N* = 16), consistent with the previously reported value of 91 nm (±8, *N* = 20) (Borst *et al*., 1985) for *T. brucei*. There are no electron-dense stripes running parallel to the plane of the *T. brucei* kDNA disc such as those observed for example in the kinetoplast of *Trypanosoma avium* (Lukes and Votypka, 2000). In the KFZ, the space between the side of the kDNA disc that faces the basal body and the inner mitochondrial membrane (Drew and Englund, 2001), another band of strongly stained fibres is clearly visible, separated from the disc by a less intensely stained gap of about 10 nm. These fibres are clearly reminiscent of the unilateral filaments that form part of the TAC. However, the unilateral filaments as described by Ogbadoyi *et al*. (2003) connect the kinetoplast disc with the inner mitochondrial membrane. The electron dense fibres observed here extend no more than ∼50 nm from the disc into the KFZ, and not as far as the membrane. We here term this structure the inner unilateral filaments, and refer to the fibres that extend from this midpoint to the membrane as the outer unilateral filaments. This distinction, based on cytochemical analysis, is corroborated by closer examination of the appearance of the unilateral filament system in kinetoplasts that were fixed and detergent extracted simultaneously (Fig. 1D) as described in Ogbadoyi *et al*. (2003). In such preparations close examination shows that the inner unilateral filaments correspond to a dense meshwork extending ∼50 nm from the disc into the KFZ, while the outer unilateral filaments subtending the detergent resistant mitochondrial membrane appear more dispersed.

**Fig. 2:**
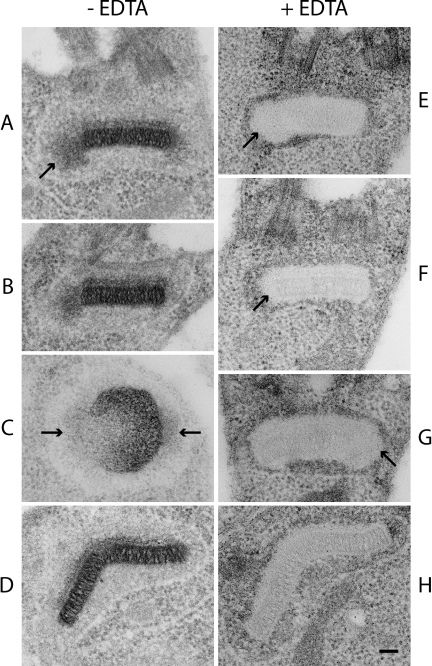
Asymmetric distribution of EDTA bleached domains around the kDNA disc A–D. Sections through kinetoplasts stained with uranyl acetate. Uranyl acetate strongly stains fibres in two domains outside the kDNA disc: lobes at the poles of some kDNA discs (marked with an arrow in A and C), and the 50 nm wide zone of inner unilateral filaments in the KFZ (A, B, D). D. Kinetoplasts that have assumed a V-configuration no longer have lobes, but the inner unilateral filaments are still visible. E–H. Sections through kinetoplasts were treated with EDTA to differentiate DNA-containing structures. EDTA bleaches the kDNA disc, the lobes (marked with an arrow in E–G) and the inner unilateral filaments (E–H). Scale bar represents 100 nm.

Further analysis confirmed the distinct nature of the inner unilateral filaments. EDTA treatment resulted in an almost complete loss of stain from the kDNA disc (Fig. 1E); often just enough contrast is maintained to discern its striate structure. However, the inner unilateral filaments are also completely bleached (Fig. 1E), but the bleached area does not extend all the way to the membrane. Strong uranyl acetate staining and sensitivity to EDTA bleaching are both indicative of DNA containing structures. Importantly, no significant bleaching occurred on the opposite face of the disc or in the mitochondrial matrix surrounding the kinetoplast or adjacent cytoplasmic ribosomes (Figs 1D and 2E–H).

In uranyl acetate stained sections, fibrous lobes are observed at the poles of some kDNA discs (Fig. 2A–C). The positioning of the lobes 180 degrees apart is clearly visible in a section cut almost parallel to the plane of the disc (Fig. 2C), where the kDNA disc is seen as a circle. These lobes are thought to represent the ultrastructural correlates of antipodal sites (Ogbadoyi *et al*., 2003) in kinetoplasts in S-phase where replicated minicircles are reattached to the kDNA network. The inner unilateral filaments are also strongly stained in these kinetoplasts with lobes, spanning the diameter of the disc but not extending over the fibrous lobes (Fig. 2A and B). The inner unilateral filaments are still clearly defined in kinetoplasts in a V configuration where the two masses of kDNA are beginning to segregate (Fig. 2D). This shows that the inner unilateral filaments remain structurally intact after kinetoplast S-phase. Following EDTA treatment, bleached lobes are observed in some kinetoplasts (Fig. 2E–G), indicating the presence of DNA in these structures. This is consistent with data showing that replicated kDNA is present at antipodal sites (Ferguson *et al*., 1992; Robinson and Gull, 1994). The lobes in the uranyl acetate stained sections extend on average 133 nm away from the disc. The bleached areas have a smaller diameter, typically around 85 nm. In kinetoplasts exhibiting a postreplicative V configuration, the inner unilateral filaments are also bleached (Fig. 2H). We applied the same cytochemical techniques to glutaraldehyde fixed *C. fasciculata* and found that EDTA bleaching also resulted in bleaching of the kDNA disc, the inner unilateral filaments and lobe structures at opposite poles of the disc (supplementary Fig. S1A–G).

We never observed any structures within the kDNA disc itself that were refractory to EDTA bleaching, therefore providing no evidence for discrete RNA processing factories within the kDNA network. It is currently an open question as to where in the trypanosome mitochondrion RNA processing and editing occurs.

Ethanolic phosphotungstic acid staining of glutaraldehyde fixed cells specifically contrasts basic proteins, and reveals structural details that are not easily discerned in uranyl acetate stained or osmicated sections (Bloom and Aghajanian, 1966; 1968).

**Fig. 3:**
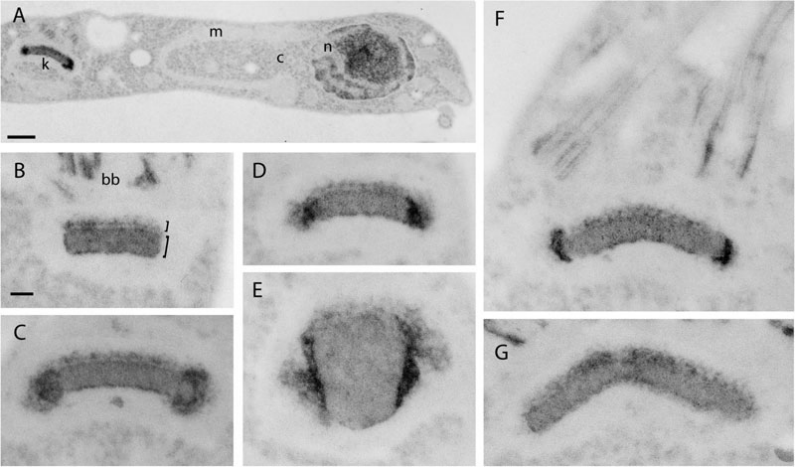
E-PTA staining for detection of basic proteins A. Longitudinal section through a *T. brucei* procyclic cell stained with E-PTA. Strong staining is observed in the nucleus (n) and the kinetoplast (k) but not in the mitochondrial matrix (m). The cytoplasm (c) stains moderately. B–G. E-PTA stained kinetoplasts at higher magnification. All images are oriented so the basal body (bb) is facing up. E-PTA staining is seen throughout the kDNA disc in all images (marked with large bracket in B). The inner unilateral filaments (marked with small bracket in B) are also stained. Strong staining of lobes is observed in some kinetoplasts (C–F). Scale bars represent 500 nm (A); 100 nm (B–G).

Figure 3A shows a longitudinal section through a procyclic *T. brucei* cell stained in this manner. The nucleus and kinetoplast are strongly contrasted and the cytoplasm shows a granular structure of intermediate contrast. The mitochondrial matrix is not contrasted. Strong binding of E-PTA to basic residues of histone proteins (Sheridan and Barrnett, 1969) accounts for the strong contrast observed in the nucleus. Lysine and arginine residues of proteins are believed to be the binding sites for the PTA, a suggestion supported by the finding that acetylation reduced PTA binding to isolated histones (Sheridan and Barrnett, 1969). In trypanosomatids, highly basic, lysine-rich proteins (KAP1-4) bind to kDNA (Xu and Ray, 1993; Xu *et al*., 1996; Hines and Ray, 1998). They are localized throughout the disc, and KAP4 was also detected at antipodal sites (Xu *et al*., 1996; Lukes *et al*., 2001). Lysine and arginine combined account for ∼20% of amino acid residues of *T. brucei* core histones and for 25–30% of residues in C. *fasciculata* KAP proteins. This suggests that the KAP proteins are good candidates for PTA binding similar to histones, and it is therefore highly likely that KAP1-4 and their homologues in *T. brucei* contribute significantly to the binding of E-PTA in kinetoplast sections. In *T. brucei*, other proteins such as Polβ-PAK (pI 10.1) (Saxowsky *et al*., 2003) could also contribute to the observed E-PTA staining in the kDNA disc.

At higher magnification (Fig. 3B–G), E-PTA staining reveals remarkable details of kinetoplast substructure. The kDNA disc itself shows intermediate contrast and the stain is more evenly distributed than uranyl acetate. In longitudinal sections, a thin rim of more concentrated E-PTA stain is often visible around the kDNA disc (Fig. 3B–D, F and G). This may indicate a higher basic protein concentration along the edge of the disc. On the side of the disc facing the basal body, E-PTA stains a fuzzy band of material that runs parallel to the face of the disc. It is ∼40 nm thick, and separated from the disc by a less intensely stained ∼10 nm gap (Fig. 3B–D and F) and thus precisely corresponds to the inner unilateral filaments described earlier. The high affinity for E-PTA of the inner unilateral filaments suggests that this structure contains significant amounts of basic proteins (probably including proteins bound to DNA), while the outer unilateral filaments do not. This provides further evidence for the distinct nature of the inner unilateral filaments which define a structurally, and likely functionally, distinct subdomain within the KFZ. E-PTA staining within this domain is still visible in kDNA discs that assume a shallow V configuration (Fig. 3F), and in a kinetoplast where the kDNA network is beginning to split in two (Fig. 3G). Scission of the kinetoplast network occurs only after gaps in minicircles have been closed after replication (Perez-Morga and Englund, 1993). It is therefore almost certain that minicircle replication has been completed in the kinetoplast in Fig. 3G, yet the appearance of the inner unilateral filaments does not change.

Ethanolic phosphotungstic acid also stains the fibrous lobes and within the lobes differentiates two subzones of different staining intensity. Adjacent to the disc there are two caps that are very strongly stained (Fig. 3C–F). In some kinetoplasts these caps are surrounded by a cloud of less densely stained material, best illustrated in Fig. 3D and E. These two images show a longitudinal (Fig. 3D) and a transverse (Fig. 3E) section through the kDNA disc respectively. The two strongly stained caps are positioned on opposite sides of the disc, 180 degrees apart. This corresponds to the positioning of antipodal sites in *T. brucei.* The difference in staining intensity within different areas of the lobes could indicate differences in proteins concentration. However, a more interesting possibility is that the subdomains visualized by E-PTA staining reflect functionally distinct regions within the antipodal sites that are populated by different enzymes. This notion is consistent with the observed spatial separation of *T. brucei* topoisomerase II and Lig kβ at antipodal sites (Downey *et al*., 2005). The lobe structures showed significant variability between individual cells. This is expected in an unsynchronized population where cells were fixed at random points in the cell cycle, and indicates that the protein composition in the antipodal sites is very dynamic. In *C. fasciculata* kinetoplasts, strong E-PTA staining was observed at the periphery of the kDNA disc and in a line through the central plane of the disc. The inner unilateral filaments were also contrasted (supplementary Fig. S1H–I). However, we did not observe any intensely stained lobe structures in *C. fasciculata*.

**Fig. 4:**
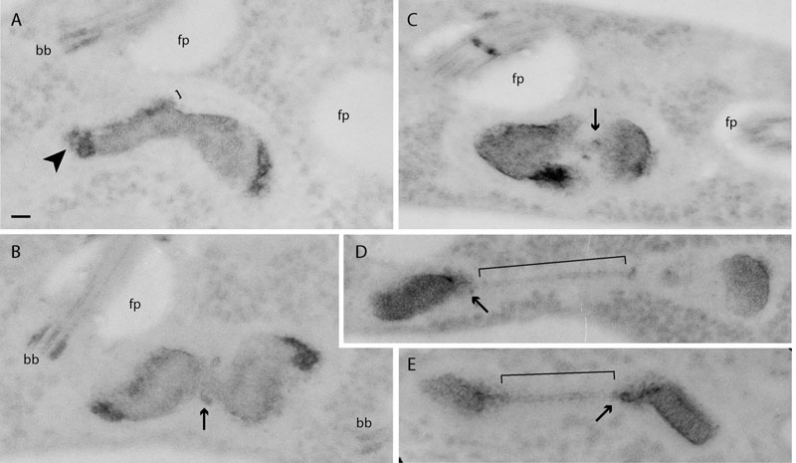
E-PTA staining of dividing kinetoplasts A–D. Showing a series of oblique sections through dividing kinetoplasts form procyclics which are ordered to show progression of division; fp: flagellar pocket, bb: basal body. (A) Basic proteins appear concentrated at the lobes (arrowhead) and the region of inner unilateral filaments (bracket). (B and C) In the centre between the two masses of kDNA, two foci of basic proteins are seen (arrow). (D) The two discs remain connected by a filament, indicated by the bracket, when they have moved a considerable distance apart. The filament emanates from a concentration of strong E-PTA staining at the periphery of the kDNA disc (arrow). E. This filament was also observed in dividing bloodstream form cells. Scale bar represents 100 nm.

Ethanolic phosphotungstic acid staining of *T. brucei* kinetoplasts reveals interesting and novel structural details of the segregation of the two replicated discs. In the rather oblique section in Fig. 4A the kDNA mass still assumes a V configuration, but an ingression between the two DNA masses is evident. The inner unilateral filaments are visible on the basal body facing side of the disc on the left, which is seen in longitudinal section, and in lobes associated with one pole of each disc. Figure 4B and C show further oblique sections through kinetoplasts where the mass of DNA has a dumbbell shape. Basic proteins are concentrated in distinct domains at the periphery of the discs. The two large foci of basic proteins that are positioned 180 degrees apart at one pole of each disc in Fig. 4B are probably derived from the antipodal sites. At the junction between the discs, there are two smaller foci of strong E-PTA staining visible on opposite sides of the DNA mass (Fig. 4B and C). In Fig. 4C, the only visible connection between the two discs is a narrow filament, indicating that we are looking at a later stage of disc segregation. Again, there are two small foci of basic proteins visible on opposite sides of the filament that connects the discs. Surprisingly, we discovered that there is still a structure that connects two kDNA discs that have moved as far as 1.3 μm apart (Fig. 4D). The nucleus of this cell is in metaphase of mitosis (as determined by kinetochores visible in the nucleus) showing that this is some time after separation of the kinetoplasts in the *T. brucei* cell cycle. This novel structure, which we termed the *nabelschnur*, consists of two parallel lines set 22 nm apart, emanating from a zone at the periphery of the disc where basic proteins appear to be concentrated. We found a similar looking structure in bloodstream form trypanosomes (Fig. 4E). Kinetoplast division as defined by the observation of dumbbell shaped kinetoplasts in DAPI stained cells is complete before mitosis in *T. brucei* (Woodward and Gull, 1990). Our finding of a structure apparently rich in basic protein(s) still connecting kinetoplasts at this late stage indicates that the final stages of kinetoplast segregation continue for some time after apparent separation of the DNA masses at the light microscope level and endures through the nuclear mitotic period.

## Discussion

The mitochondrial genome in kinetoplastids is organized into an unusually complex structure. The architecture of the kinetoplast is crucial for replication of the concatenated mass of mini and maxicircles. Enzymes involved in the replication of minicircles are located in distinct, precisely positioned domains and the topological relationship between these domains is an integral part of the current model of minicircle replication (Liu *et al*., 2005). Moreover, the structural link of the kinetoplast with the basal body provides the mechanism that ensures that each daughter cell inherits one kinetoplast at cell division. The importance of domain architecture for other processes (transcription, RNA processing and editing) has not been explored. Our cytochemical analysis provides new information on the ultrastructural organization of the *T. brucei* kinetoplast, and specifically the KFZ. The role of the KFZ as a compartment with specific functions both in minicircle replication and kinetoplast segregation has been highlighted in recent studies (Drew and Englund, 2001; Ogbadoyi *et al*., 2003). The KFZ contains the unilateral filaments that form a part of the TAC, which physically connects the kinetoplast to the basal body in *T. brucei, T. cruzi, C. fasciculata* (Souto-Padron *et al*., 1984; Ogbadoyi *et al*., 2003) and probably in other kinetoplastids. The KFZ is also the space where free minicircles are replicated (Drew and Englund, 2001). This was determined in *C. fasciculata* by fluorescence *in situ* hybridization using probes specific for the minicircle l-strand, which under non-denaturing conditions are specific for replication intermediates. Proteins involved in minicircle replication have been demonstrated by immunofluorescence microscopy to localize to the KFZ, including the *C. fasciculata* universal minicircle sequence-binding protein UMSBP (Abu-Elneel *et al*., 2001) and *T. brucei* DNA polymerases POLIB and POLIC (Klingbeil *et al*., 2002). Our description of the asymmetric distribution of DNA domains surrounding the *T. brucei* and *C. fasciculata* kDNA discs described here suggests that the KFZ also plays an important role in kDNA metabolism.

**Fig. 5:**
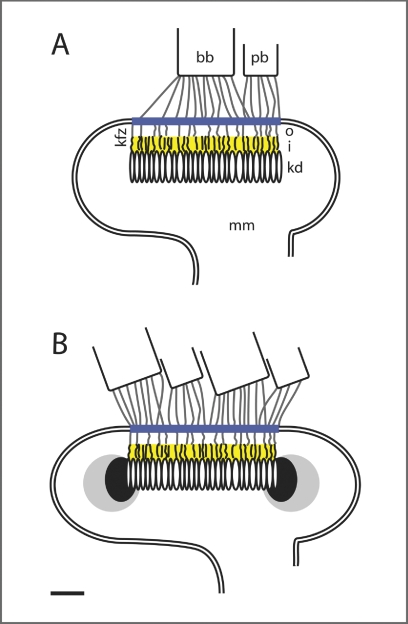
Kinetoplast domain architecture A. Drawing of a longitudinal section through a *T. brucei* kinetoplast indicating the distinction between the inner and outer unilateral filaments of the TAC. The unilateral filaments physically connect the kDNA disc (kDa) to a specialized region of the mitochondrial membrane (bold blue lines). The inner unilateral filaments (i) adjoin the basal body facing side of the kDNA disc, and span a ∼50 nm wide subdomain of the KFZ (highlighted in yellow). TEM cytochemistry indicates that this domain contains DNA and basic proteins. The outer unilateral filaments (o) extend beyond this subdomain and form a connection with the membrane. B. Replicating kinetoplasts are characterized by fibrous lobes at the two poles of the kDNA disc (grey circles), representing the antipodal sites where replicated DNA is reattached to the network. These structures are rich in basic proteins and are composed of distinct subdomains (indicated by the black circles). (bb) basal body; (pb) probasal body; (mm) mitochondrial matrix. Scale bar represents 100 nm.

The unilateral filaments as defined by Ogbadoyi *et al*. (2003) extend from the kDNA disc to the inner mitochondrial membrane, transversing the entire KFZ. The molecular composition of these fibres is unknown. Here we provide for the first time insight into the chemical nature of unilateral filaments and show that they are separated into at least two compositionally distinct zones. The inner unilateral filaments located proximal to the kDNA disc are characterized by their strong reactivity with uranyl acetate and E-PTA, and sensitivity to EDTA bleaching (i.e. complete loss of uranyl acetate stain within 15 min). Such a staining profile is essentially similar to that of the kDNA disc itself and nuclear chromatin and indicates that DNA and basic proteins are likely major constituents of the inner unilateral filaments. We recognize that, while both staining procedures have been shown be specific for the respective characterized component, it is likely that the resulting pattern emerges from detection of multiple complexes containing the DNA or basic protein component. Further dissection of the inner unilateral filament region will require complementary biochemical and reverse genetic approaches. The filaments observed connecting to the mitochondrial membrane (Ogbadoyi *et al*., 2003), now defined here as outer unilateral filaments, are less intensely stained by uranyl acetate, and completely refractory to EDTA bleaching and E-PTA staining. Their identity remains to be explored. Figure 5 shows a schematic overview of kinetoplast domain architecture and the organization of unilateral filaments within the KFZ.

Association of the TAC with the kDNA disc establishes an asymmetry with regard to the two faces of the disc. This notion is corroborated by the cytochemical staining presented here, which indicates the presence of DNA and basic proteins within the zone of unilateral filaments and at antipodal sites, but not on the opposite face of the disc. The unilateral filaments could play a role in organizing and directing structural features of kDNA replication (Ogbadoyi *et al*., 2003) and may prevent free minicircles from diffusing into other areas of the mitochondrial matrix. Thus far, the ultrastructural features revealed by our cytochemical staining are consistent with the current model of kDNA replication.

Intriguingly, however, our data strongly suggest that DNA is present in the KFZ throughout the cell cycle. This is not predicted by the current model of kDNA replication. If minicircles are released into the KFZ only during replication, then why do we see persistence of this structure throughout the cell cycle? The FISH probe used to detect minicircle replication intermediates in the *C. fasciculata* KFZ specifically detected single stranded DNA and would not detect double stranded DNA. Interestingly, a weak FISH signal was detected in all cells (Drew and Englund, 2001). Our findings raise the question of what the nature of the DNA filaments in the *T. brucei* KFZ is, whether they are linked to the network, and most importantly, what they are doing in the KFZ when not undergoing replication. It is currently unknown how other processes such as transcription and editing of RNA are organized within the mitochondrial space and in relation to the kDNA network. It has been suggested that proximity of mini and maxicircles may facilitate RNA editing, thus accounting for the complex structure of the kDNA (Shapiro and Englund, 1995). Whether RNA editing occurs in stable RNPs and where these might be assembled is an open question (Madison-Antenucci *et al*., 2002). The size of the 20S editosome that contains the core enzymatic activities capable of catalysing one round of RNA editing *in vitro* has been calculated to be ∼600 kDa (Rusche *et al*., 1997), and additional proteins associated with the editosome have since been discovered (Simpson *et al*., 2004). While the size of kinetoplast editing complexes *in vivo* is not known, RNPs of ≥ 600 kDa are expected to be large enough to be within the limits of resolution of TEM. Using Bernhard's EDTA regressive stain, we therefore specifically looked for clusters of ribonucleoprotein particles or ‘RNA factories’ within the kDNA disc but did not find any. We never observed any structures refractory to EDTA bleaching similar to the 40 nm granules reported to be ‘preferentially located at the two extremities of the kinetoplast’ of *T. cruzi* (Esponda *et al*., 1983). In contrast, the areas immediately adjacent to the bleached kDNA disc and inner unilateral filaments were completely refractory to EDTA bleaching, possibly indicating the presence of RNPs in these areas. We can speculate that the domain defined by the inner unilateral filaments may be the site of transcription, and RNA editing may occur in the adjacent areas. This could be tested in the future by determining at an EM level of resolution the subcellular localization of RNA polymerases and proteins with a role in RNA processing for example. To date few proteins involved in RNA editing have been localized *in situ*. Mitochondrial localization has been demonstrated for MRP1/gBP21 and the RNA ligases KREL1/TbMP52 and KREL2/TbMP48 (Allen *et al*., 1998; McManus *et al*., 2001; Panigrahi *et al*., 2001). The signal for MRP1 was most intense in the kinetoplast (Allen *et al*., 1998).

Ethanolic phosphotungstic acid staining has been used in several studies to analyse the distribution of basic proteins *T. cruzi* (Souto-Padron and De Souza, 1978; 1979; Esponda *et al*. 1983; Rocha *et al*., 2006). In the *T. cruzi* kinetoplast, E-PTA stains a broad rim around the periphery of the kDNA disc. In addition, a line through the central plane of the kDNA disc is visible in some images (for example, fig. 10 in Souto-Padron and De Souza, 1978; fig. 5E in Rocha *et al*., 2006). Strong staining of antipodal sites or structures associated with dividing kinetoplasts have not been reported. The E-PTA staining pattern we observed in the *T. brucei* kinetoplast differs from these images. Staining always appeared homogenous throughout the disc, with only a thin rim of more concentrated stain at the periphery. The inner unilateral filaments were strongly contrasted, and in some images, antipodal sites were very electron dense, revealing details of substructure (Fig. 3). By contrast, the E-PTA staining pattern we observed in *C. fasciculata* kinetoplasts (Fig. S1) more closely resembles the published images of *T. cruzi* epimastigote kinetoplasts. It is interesting to note that the position of the electron dense line through the central plane of the *C. fasciculata* disc revealed by E-PTA staining (Fig. S1) coincides with the central electron dense line observed after disruption of the KAP1 gene (Lukes *et al*., 2001).

A number of issues of kDNA organization remain unresolved including how the replicated DNA network is split in two and how the daughter kinetoplasts are remodelled. We conclude from the E-PTA staining pattern that clusters of basic proteins remained associated with the periphery of the discs throughout the segregation process. Such proteins may have an active role in catalysing the final steps of network segregation. Alternatively, persistence of domains with a role in replication, such as the antipodal sites (Johnson and Englund, 1998) may be necessary to transmit spatial information about kinetoplast domains to daughter cells. Our study shows that there are additional events in the final stages of kinetoplast segregation that are not yet incorporated into models of kinetoplast replication and segregation. Our discovery of a filament apparently rich in basic proteins that connects two kDNA discs that have moved > 1 μm apart was unexpected. It will be of interest to identify these proteins and their role in kinetoplast segregation. Is this a physical structure that orchestrates the physical separation of the kDNA networks analogous to the separation of chromosomes by the mitotic spindle? Alternatively one might envisage a function for this filament in positioning the kinetoplast while division of the mitochondrion takes place. The discovery of this novel structure indicates that even when one sees the masses of kDNA separated, for example in a DAPI stained trypanosome, kinetoplast division is not necessarily complete. This discovery has implications for analysis and understanding the RNAi studies of mutant phenotypes and therefore the function of mitochondrial proteins. At which point the *nabelschnur* is finally cut remains to be determined, but we expect that it occurs in precise co-ordination with other events of the trypanosome cell cycle.

## Experimental procedures

### Cells

*Trypanosoma brucei* 427 procyclics were grown *in vitro* at 28°C in SDM 79 medium (Brun and Schonenberger, 1979); bloodstream forms were grown *in vitro* at 37°C with 5% CO_2_ in HMI-9 medium supplemented with 15% FCS (Hirumi and Hirumi, 1989).

### Electron microscopy

For cytochemical analysis, cells in mid-log phase of growth (3–8 × 10^6^ cells ml^−1^) were fixed in 2.5% glutaraldehyde in 0.1 M phosphate buffer pH 7.0 for 2 h at room temperature, without osmium tetroxide postfixation. Cells were dehydrated in a graded series of ethanol and embedded in epon resin. Thin sections were examined in an FEI Tecnai 12 transmission electron microscope. Extraction of cells with detergent during fixation and subsequent preparation for TEM was done as described by Ogbadoyi *et al*. (2003).

### Cytochemistry

For staining of basic proteins with E-PTA, cells were fixed as described above. During the final dehydration step, the material was incubated for 2 h in 2% phosphotungstic acid in 100% ethanol, and washed in 100% ethanol before embedding in resin. Thin sections were examined without further processing.

Bernhard's EDTA regressive staining was performed essentially as described (Bernhard, 1969; Moyne, 1980). Thin sections (silver interference colour) were stained with 5% aqueous uranyl acetate for 5 min and then washed in water for at least 5 min. For regressive staining, sections were floated on a drop of 0.2 M EDTA, pH 7.0 for 15–60 min. Maximal bleaching of kDNA was observed within 15 min. As controls, sections treated with EDTA were restained with uranyl acetate to confirm that the reaction was reversible. Prior to examination, both uranyl acetate-only stained and EDTA treated sections were stained for 1 min with 0.1% lead citrate.

## Supplementary material

The following supplementary material is available for this article:

Fig. S1 Analysis of *C. fasciculata kinetoplasts. C. fasciculata* were harvested from log phase cultures and prepared for TEM as detailed in Experimental Procedures. (A-D) *C. fasciculata* stained with uranyl acetate. The kDNA disc (kd) and inner unilateral filaments (i) are strongly contrasted. The width of the disc is 271 nm (±16.4, N = 26) and the inner unilateral filaments extend 44 nm (±9.1, N = 23) into the KFZ. Lobe structures are visible at opposite poles of the discs in C and D. The lobes have a dense core adjacent to the disc (arrowhead), surrounded by a cloud of more diffuse staining. (E–G) Treatment with EDTA removes uranyl acetate from the nuclear chromatin (ch), the kDNA disc (kd) and the inner unilateral filaments (i), resulting in a bleached appearance of these structures. Bleached lobes are visible at opposite poles of the discs in E and F (arrowheads). (H–I) *C. fasciculata* cells stained with E-PTA. Staining is most intense at the periphery of the kDNA disc (kd) and in a line through the central plane of the disc (arrows). The inner unilateral filaments (i) are also slightly contrasted. No E-PTA staining is observed in the mitochondrial matrix (m). Scale bar in A, F and H represents 500 nm. Scale bar in B represents 100 nm.

This material is available as part of the online article from:

http://www.blackwell-synergy.com/doi/abs/10.1111/j.1365-2958.2007.05749.x

(This link will take you to the article abstract).
