# Supplementary figures and images for: Structural asymmetry and discrete nucleic acid subdomains in the Trypanosoma brucei kinetoplast
Source: Mol Microbiol. 2007 Jun 1;64(6):1529–39. doi: 10.1111/j.1365-2958.2007.05749.x (PMC1974780; doi:10.1111/j.1365-2958.2007.05749.x)

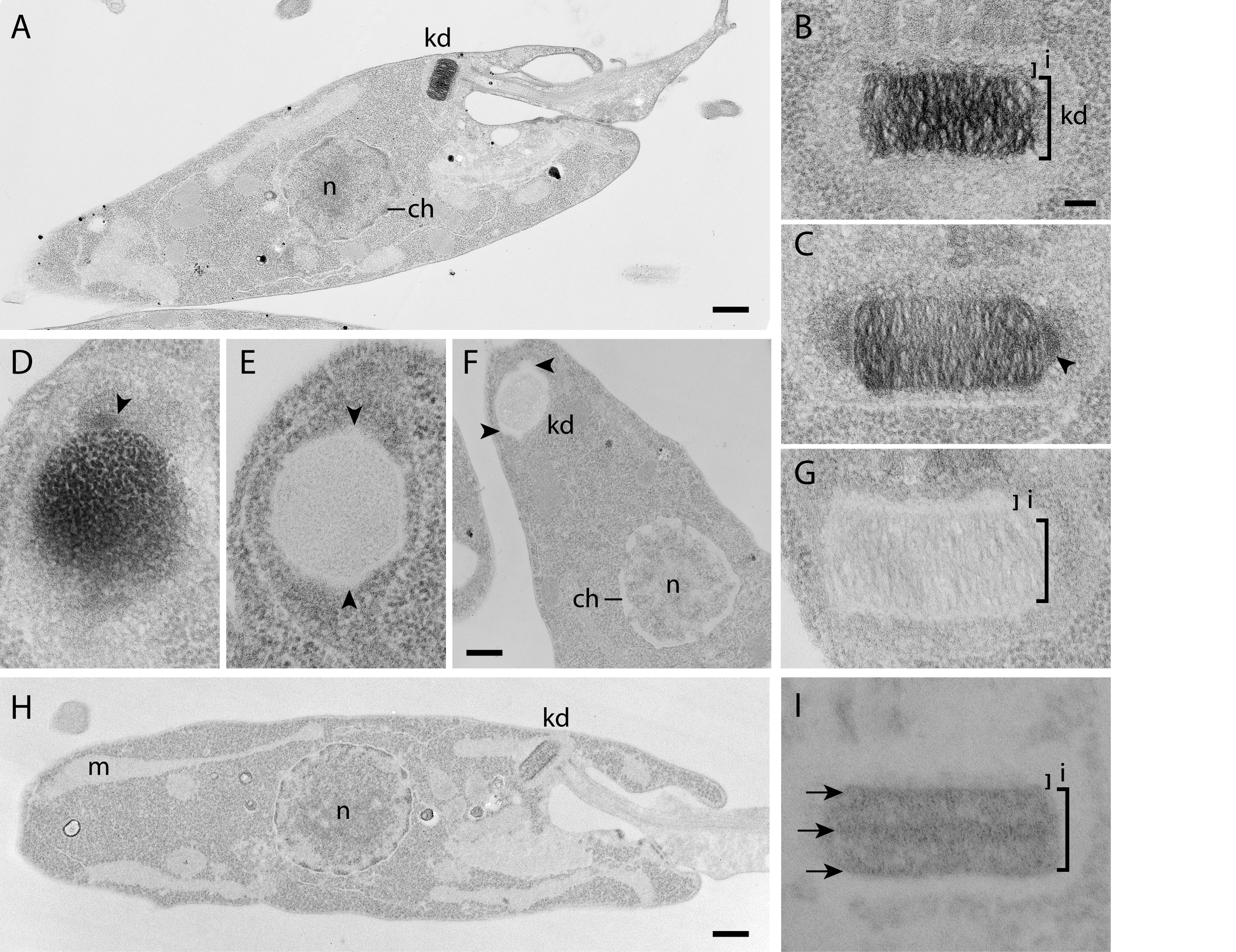

Supplement: Fig. S1 — Analysis of C. fasciculata kinetoplasts. C. fasciculata were harvested from log phase cultures and prepared for TEM as detailed in Experimental Procedures. (A-D) C. fasciculata stained with uranyl acetate. The kDNA disc (kd) and inner unilateral filaments (i) are strongly contrasted. The width of the disc is 271 nm (±16.4, N = 26) and the inner unilateral filaments extend 44 nm (±9.1, N = 23) into the KFZ. Lobe structures are visible at opposite poles of the discs in C and D. The lobes have a dense core adjacent to the disc (arrowhead), surrounded by a cloud of more diffuse staining. (E–G) Treatment with EDTA removes uranyl acetate from the nuclear chromatin (ch), the kDNA disc (kd) and the inner unilateral filaments (i), resulting in a bleached appearance of these structures. Bleached lobes are visible at opposite poles of the discs in E and F (arrowheads). (H–I) C. fasciculata cells stained with E-PTA. Staining is most intense at the periphery of the kDNA disc (kd) and in a line through the central plane of the disc (arrows). The inner unilateral filaments (i) are also slightly contrasted. No E-PTA staining is observed in the mitochondrial matrix (m). Scale bar in A, F and H represents 500 nm. Scale bar in B represents 100 nm. [file mmi0064-1529-fs1.tif]
